# Supplementary material for: Dynamic Changes in Microvascular Density Can Predict Viable and Non-Viable Areas in High-Risk Neuroblastoma
Source: Cancers (Basel). 2023 Feb 1;15(3):917. doi: 10.3390/cancers15030917 (PMC9913651; doi:10.3390/cancers15030917)
Supplement: Supplementary file 1 [file cancers-15-00917-s001.zip › cancers-2151377-supplementary.pdf]

Supplementary Materials

Table S1. Table of the values obtained from the systematic uniform random sampling performed to establish the representative amount of regions of interest (ROIs) per patient. Three consecutive values after the 5th ROI were within the 95% confidence interval of the standard error of the mean.

Regions of interest (ROI)	Mean Vascular density	Average	Standard Error	95% Confidence Interval
ROI 1	0.007	0.006	0.0015	[0.003 – 0.008]
ROI 2	0.004	0.006	0.0009	[0.004 – 0.007]
ROI 3	0.006	0.011	0.0056	[0 – 0.022]
ROI 4	0.028	0.010	0.0045	[0.001 – 0.019]
ROI 5	0.006	0.010	0.0037	[0.002 – 0.017]
ROI 6	0.007	0.009	0.0033	[0.002 – 0.015]
ROI 7	0.003	0.013	0.0049	[0.003 – 0.022]

Table S2. Summary of the histological subtype for each patient included in the study before and after treatment.

Patient ID	Histological subtype pre-chemotherapy	Histological subtype post-chemotherapy
Patient 1	Undifferentiated	Differentiated
Patient 2	Undifferentiated	Poorly Differentiated
Patient 3	Undifferentiated	Non Viable
Patient 4	Undifferentiated	Ganglioneuroblastoma
Patient 5	Undifferentiated	Non Viable
Patient 6	Undifferentiated	Poorly Differentiated
Patient 7	Undifferentiated	Non Viable
Patient 8	Undifferentiated	Differentiated
Patient 9	Undifferentiated	Poorly Differentiated
Patient 10	Undifferentiated	Poorly Differentiated
Patient 11	Poorly Differentiated	Non Viable
Patient 12	Poorly Differentiated	Undifferentiated
Patient 13	Poorly Differentiated	Poorly Differentiated
Patient 14	Poorly Differentiated	Poorly Differentiated
Patient 1	Undifferentiated	Differentiated
Patient 2	Undifferentiated	Poorly Differentiated
Patient 3	Undifferentiated	Non Viable
Patient 4	Undifferentiated	Ganglioneuroblastoma
Patient 5	Undifferentiated	Non Viable
Patient 6	Undifferentiated	Poorly Differentiated
Patient 7	Undifferentiated	Non Viable
Patient 8	Undifferentiated	Differentiated
Patient 9	Undifferentiated	Poorly Differentiated
Patient 10	Undifferentiated	Poorly Differentiated
Patient 11	Poorly Differentiated	Non Viable
Patient 12	Poorly Differentiated	Undifferentiated
Patient 13	Poorly Differentiated	Poorly Differentiated
Patient 14	Poorly Differentiated	Poorly Differentiated
Patient 15	Poorly Differentiated	Differentiated
Patient 16	Poorly Differentiated	Differentiated
Patient 17	Poorly Differentiated	Differentiated
Patient 18	Poorly Differentiated	Poorly Differentiated
Patient 19	Poorly Differentiated	Ganglioneuroblastoma
Patient 20	Poorly Differentiated	Ganglioneuroblastoma
Patient 21	Differentiated	Differentiated
Patient 22	Differentiated	Undifferentiated
Patient 23	Differentiated	Poorly Differentiated
Patient 24	Differentiated	Differentiated
Patient 25	Differentiated	Differentiated
Patient 26	Differentiated	Differentiated
Patient 27	Differentiated	Differentiated
Patient 28	Differentiated	Differentiated
Patient 29	Differentiated	Poorly Differentiated
Patient 30	Differentiated	Ganglioneuroblastoma

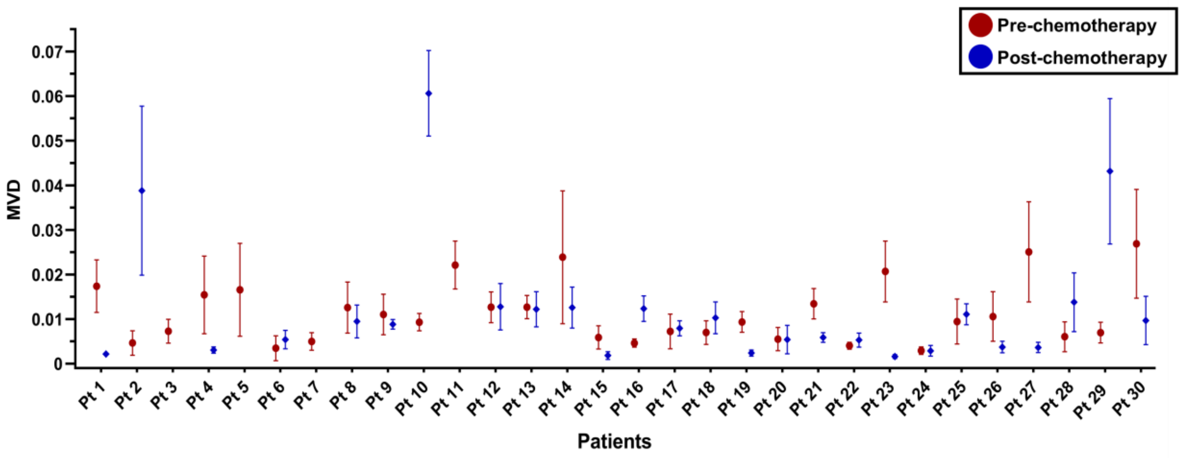

Figure S1. Bars showing mean and standard error of the MVD for each patient before (red) and after induction chemotherapy (blue).

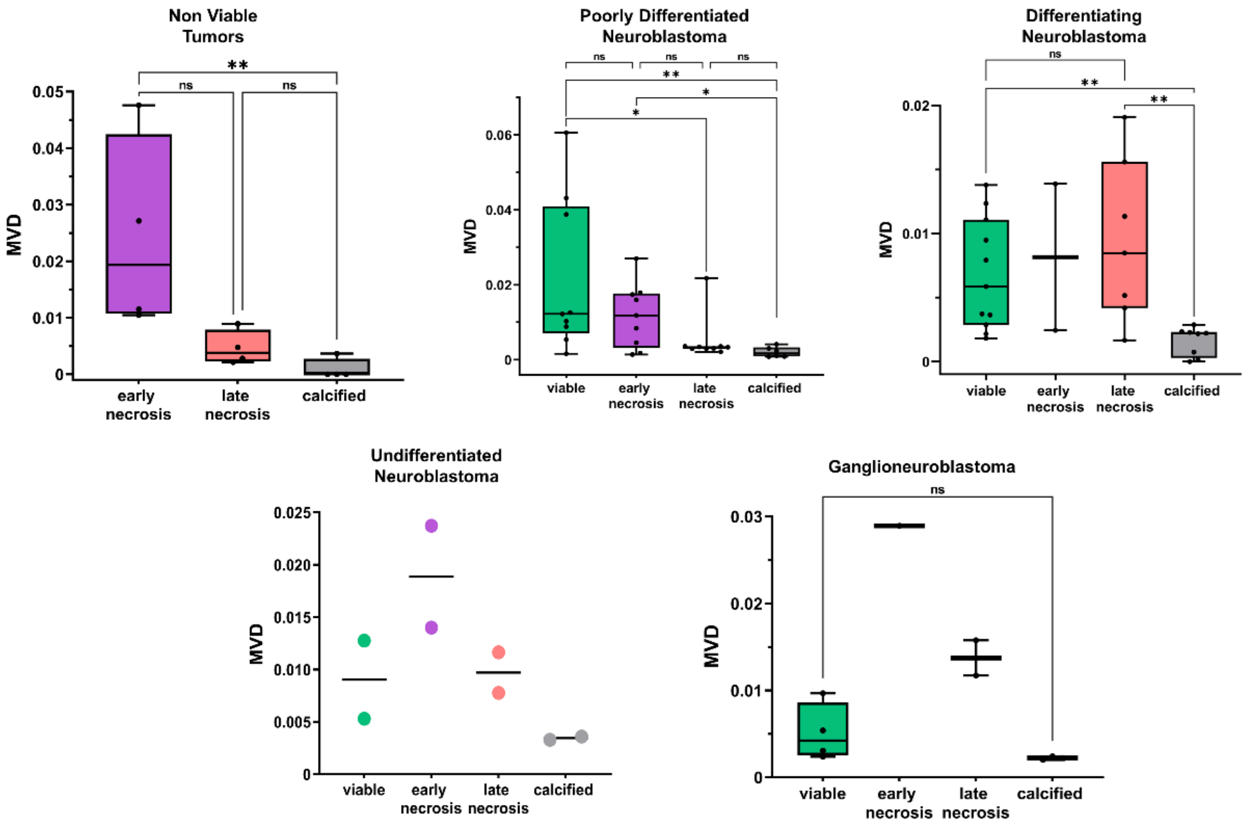

Figure S2. Boxplot showing the distribution of patients MVD grouped by histological subtype in viable and non-viable regions after induction chemotherapy. Box represents inter-quartile range. Bars rep-resent 5—95% range. *p<0.05; **p<0.01
